# Study on depressive symptoms in patients with Mayer-Rokitansky-Küster-Hauser syndrome: an analysis of 141 cases

**DOI:** 10.1186/s13023-020-01405-9

**Published:** 2020-05-24

**Authors:** Na Chen, Shuang Song, Yanping Duan, Jia Kang, Shan Deng, Hongxin Pan, Lan Zhu

**Affiliations:** 1 Department of Obstetrics and Gynecology, Peking Union Medical College Hospital, Peking Union Medical College, Chinese Academy of Medical Sciences, No. 1 Shuaifuyuan, Beijing, 100730 China; 2 Department of Psychology, Peking Union Medical College Hospital, Peking Union Medical College, Chinese Academy of Medical Sciences, Beijing, 100730 China; 3 Department of Obstetrics and Gynecology, The 3rd Affiliated Hospital of Shenzhen University, Luohu Hospital, Shenzhen, Guangdong China

**Keywords:** MRKH syndrome, Depression, Depressive symptom, Risk factor

## Abstract

**Background:**

Mayer-Rokitansky-Küster-Hauser (MRKH) syndrome is a rare congenital disease characterized by uterovaginal agenesis. The diagnosis of MRKH syndrome generally leads to considerable emotional burdens on patients. However, studies focusing on the psychological influence on patients are limited and of unsatisfactory quality. The aim of this study was to investigate the prevalence of depressive symptoms in Chinese patients with MRKH syndrome and to identify the factors associated with depressive symptoms. We recruited 141 patients with MRKH syndrome and 178 age-matched healthy women as control group in this cross-sectional study. Depressive symptoms were assessed by the Patient Health Questionnaire-9 (PHQ-9). Other parameters such as sociodemographic characteristics, treatment histories, personality traits, and attitudes toward femininity and offspring, were also gathered in the self-administered questionnaire.

**Results:**

The PHQ-9 score was significantly higher in MRKH patient group than the age-matched control group (7.0 (4.5–11.0) vs. 6.0 (3.0–9.0)), median and IQRs) (*P* = 0.015). A total of 75.2% of MRKH patients suffered from depressive symptoms, and 34.0% reached a moderate to severe level, while the proportion in the control group was 61.2 and 24.2% respectively. Compared with other age groups, patients in their 20s scored higher on the PHQ-9. Patients with higher neuroticism levels in personality traits (OR 1.19 95% CI 1.11–1.28), negative self-evaluation of femininity (OR 3.964, 95% CI 1.371–11.464) and sexual dysfunction (OR 4.81 95% CI 1.24–18.72) (compared with those having no sexual activity) were more likely to exhibit depressive symptoms.

**Conclusions:**

Three-quarters of MRKH patients show depressive symptoms, and one-third of these individuals are even at risk for depressive disorders. Therefore, depressive symptom screening and proper psychotherapy in MRKH patients are of great importance.

## Background

Mayer-Rokitansky-Küster-Hauser (MRKH) syndrome is a congenital malformation characterized by the absence of uterus and the upper two-thirds of the vagina, with an incidence of 1 in 4000–5000 females [1]. Patients with MRKH syndrome have a normal karyotype and display regular female secondary sexual characteristics. Nevertheless, the anatomical abnormality leads to sexual intercourse difficulties and infertility, which is a considerable emotional burden, causing questions of identity, confusion in social and sexual roles, and the development of negative self-beliefs [2, 3]. The most common first symptom of MRKH syndrome is primary amenorrhea. Therefore, a majority of cases are diagnosed during adolescence, a developmental period when adult body image and sexual identity emerge. The diagnosis of MRKH syndrome will cause various identity issues and aggravate emotional burdens in this sensitive age [4].

Due to emotional burdens caused by the diagnosis, management and treatment of MRKH syndrome, depressive symptoms appear frequently. Patients have reported depressive emotions, even suicidal thoughts, in reaction to the diagnosis of this condition [5, 6]. While living with this disease, depression tendencies also exist [3].

Although there are large psychological impacts on patients with MRKH syndrome, studies focusing on the state of depression in MRKH patients are quite limited. The sample size in published studies is relatively small. With an incidence of 1 in 4000–5000 females, there are about 140,000–170,000 patients with MRKH syndrome in China. Yet, no study on the psychological assessment of Chinese MRKH patients was published till now. The aim of our study was to screen the depressive symptoms of Chinese patients with MRKH syndrome by investigating the prevalence of depressive symptoms and identifying factors associated with these symptoms.

## Material and methods

### Participants and procedures

This study was a cross-sectional, web-based questionnaire survey. All the MRKH patients having an office visit in the Center of Obstetrics and Gynecology, Peking Union Medical College Hospital (PUMCH) from January 2018 to December 2018 were invited to join this survey. The introductory letter explaining the purpose and voluntary nature of the study and the link to the online questionnaire were distributed. Patients willing to participate in this survey completed an anonymous questionnaire and submitted their answers on the web server. We excluded patients with uncertain diagnosis of MRKH syndrome, patients under 16 years old and patients who already had pre-existing psychiatric diagnoses before the diagnosis of MRKH syndrome. A total of 218 questionnaires were distributed, and 141 valid and completed questionnaires were retrieved. The valid response rate was 64.7%.

Control-group women were recruited from the Physical Examination Center of PUMCH. A link of similar online questionnaire (without information on disease and treatment) was distributed to women interested in participating the study. Of 240 control-group women who were approached, 178 returned completed questionnaires (response rate: 74.2%). The study was approved by the Ethics Committee of PUMCH (Project number: S-K471).

### Measures

Patient demographic characteristics, including age, residence, religious belief, education level, marital status, the state of sexual partners and offspring, family support and relationship, were all gathered in the questionnaire. Disease related information consisted of the age of diagnosis and treatment received.

The Patient Health Questionnaire-9 (PHQ-9) was used to screen the depressive symptoms of the respondents. PHQ-9 is a depression-screening instrument widely used in nonpsychiatric settings. The Chinese version of PHQ-9 is validated in the general population in China [7]. This analysis is a 9-item self-administered questionnaire containing questions that reflect all 9 criteria upon which the diagnosis of Diagnostic and Statistical Manual of Mental Disorders, 4th Edition (DSM-IV) depressive disorder is based [8]. This questionnaire scores each item as 0 to 3. Using the summed-item scoring method, the PHQ-9 score is divided into several categories of increasing severity in most analyses: no (0–4), mild [5–9], moderate [10–14], moderately severe [15–19] and severe (≥20, 8). Hence, people who get 5 points or higher in PHQ-9 were defined as having depressive symptoms. Based on the results of a meta-analysis, a cutoff point of ≥10 has a pooled sensitivity of 0.77 and a specificity of 0.85 to detect major depressive disorder, representing a satisfactory diagnostic performance for screening purposes [9]. Therefore, we adopted a cutoff point of 10 in this survey, dividing patients into depressive and nondepressive groups.

In addition to screening depression symptoms, other measures were also collected for further analysis: 1) The Eysenck Personality Questionnaire-Revised Short Scale for Chinese (EPQ-RSC) was used to measure the personality traits of patients in this study. This questionnaire was developed by Eysenck in 1985 and translated and revised by Qian et al. in 2000 [10]. The EPQ-RSC measures personality traits from 4 dimensions: extraversion (E), neuroticism (N), psychoticism (P) and a lie detector inventory (L). Each dimension contains 12 true-false items. The EPQ-RSC has been proven to have good psychometric properties among individuals above 16 years old in the general population. 2) The Chinese Version of the Female Sexual Function Index (CVFSFI) is a brief, multidimensional scale for assessing sexual function in women. The CVFSFI comprises 19 items that measure 6 domains of sexual dysfunction, including arousal, orgasm, desire, lubrication satisfaction and pain [11]. In this study, all women who were sexually active (including the non-treated patients and patients who had received the construction of neovagina) in the past 4 weeks were required to complete the CVFSFI. Those who have no relevant sexual activity in the past 4 weeks could skip the questionnaire. Based on epidemiological research on Chinese women, the cutoff score for the Female Sexual Function Index (FSFI) total score was 23.45 [12]. Higher scores indicate better sexual function. Based on the CVFSFI questionnaire, patients were divided into having no sexual activity, sexual dysfunction (score of CVFSFI< 23.45) and normal sexual function (score of CVFSFI≥23.45) groups. 3) Questions, such as “Do you think you possess good femininity?” and “What’s your plan for generating offspring?”, were also included in the questionnaire to evaluate the attitudes of the participants toward their female image and next generation offspring, which are potentially impacted by their diseases.

### Data analysis

Descriptive statistics were presented as the mean values±standard deviations (SD) or medians and interquartile ranges (IQRs) for continuous variables and frequencies for categorical variables. The mean ± SD only was computed when data followed a Gaussian distribution otherwise the medians and IQRs were reported instead. The main outcome was depressive symptoms. The analysis of variance (ANOVA) methods were applied to assess the depressive scores among different age groups. Univariate analysis was used to screen variables associated with outcomes. Then, baseline variables that were considered clinically relevant or factors with a *P* < 0.1 in univariate analysis were enrolled in a stepwise multiple logistic regression analysis to identify the potential risk factors for depressive states in patients with MRKH syndrome. Pearson’s χ^2^ test or Fisher’s exact test were used to analyze categorical variables, and the independent sample t-test or the Mann-Whitney U test were performed to analyze continuous variables. *P* < 0.05 was considered statistically significant. Moreover, Bonferroni’s correction was used for pairwise comparison. All statistical analyses were performed by using IBM®SPSS® 21.0 statistical package (SPSS Inc., Chicago, Illinois, USA).

## Results

Table 1 shows the demographic features, treatment histories and psychological results of the investigated patients. The mean age was 25.78 ± 4.62 yrs. for the patient group and 26.04 ± 5.07 yrs. for the control group (*P* = 0.729). The mean years since diagnosis was 7.72 ± 4.50. Among 141 patients with MRKH syndrome, most had no religious belief (90.1%), lived in urban areas (61.0%), received an education level above college (73.0%), were single (78.7%), had a good family relationship (80.9%) and could gain support from family after diagnosis (90.1%). Half of them (49.6%) had sexual relationships. Only 3 (2.1%) investigated patients had their children by adoption or surrogacy. Regarding the treatment option, 33 (23.4%) patients did not receive any treatment, and 67 (47.5%) patients received nonsurgical dilation, while the other 41 (29.1%) patients underwent vaginoplasty surgery. Of these 41 patients who underwent surgical vaginoplasty, 29 patients received vaginoplasty with tissue-engineered biomaterial grafts and the remaining 12 patients received laparoscopic peritoneal vaginoplasty. In terms of attitude toward femininity and offspring, 91 (64.5%) patients thought that they possessed good femininity, while the other patients denied this statement. Most patients (79.4%) showed a willingness to obtain offspring. Compared to adoption (25.5%), more patients were in favor of surrogacy (53.9%).

**Table 1:** The demographic features, treatment history and psychological results of patients with MRKH syndrome

Variables	All patients with MRKH syndrome (*n* = 141)	Depressive symptoms		
		Depressive group (*n* = 48)	Non-depressive group (*n* = 93)	*P* value
Age	25.78 ± 4.62	25.23 ± 4.08	26.06 ± 4.88	0.311
Years since diagnosis	7.72 ± 4.50	7.35 ± 3.77	7.91 ± 4.84	0.725
Religious belief				0.663
Yes	14 (9.9%)	6	8	
No	127 (90.1%)	42	85	
Residence				0.407
Urban	86 (61.0%)	27	59	
Rural	55 (39.0%)	21	34	
Offspring				0.521
Yes (Adoption or surrogacy)	3 (2.1%)	0	3	
No	138 (98.1%)	48	90	
Family relationship				0.085
Good	114 (80.9%)	35	79	
Average	27 (19.1%)	13	14	
Support from family after diagnosis				0.374
Yes	127 (90.1%)	43	84	
No	14 (9.9%)	5	9	
Educational level				0.043*
≤ Secondary school	38 (27.0%)	18	20	
≥ College	103 (73.0%)	30	73	
Marital status				0.226
Single	111 (78.7%)	35	76	
Married	30 (21.3%)	13	17	
Sexual partner				0.768
Yes	70 (49.6%)	23	47	
No	71 (50.4%)	25	46	
Treatment				0.748
Yes	108 (76.6%)	36	72	
No	33 (23.4%)	12	21	
Positive self-evaluation on femininity				< 0.001*
Yes	91 (64.5%)	21	70	
No	50 (35.5%)	27	23	
Plan about offspring				0.620
Yes	112 (79.4%)	37	75	
No	29 (20.6%)	11	18	
Female sexual dysfunction				< 0.001*
Yes	28 (19.8%)	18	10	
No	42 (29.8%)	7	35	
Not sexually active	71 (50.4%)	25	46	
EPQ-RSC				
EPQ-P	49.21 ± 8.84	51.85 ± 8.56	48.02 ± 8.72	0.016*
EPQ-E	49.49 ± 11.25	45.34 ± 11.87	51.58 ± 10.32	0.005*
EPQ-N	59.39 ± 11.58	68.88 ± 6.49	54.77 ± 10.70	< 0.001*
EPQ-L	50.47 ± 9.46	49.31 ± 8.76	51.13 ± 9.56	0.134

### Prevalence of depressive symptoms in the patient group and control group

**Fig. 1:**
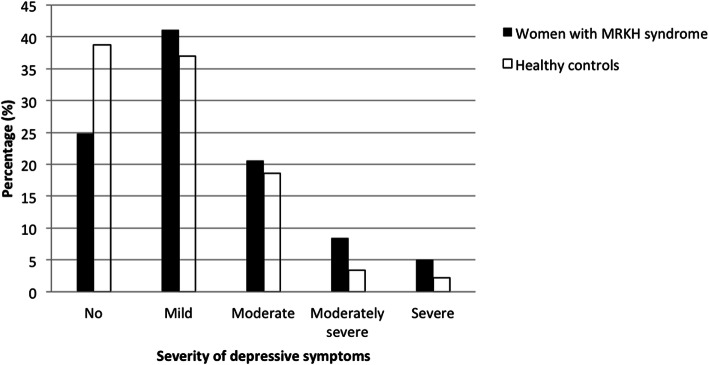
Severity of depressive symptoms in patients with MRKH syndrome and healthy controls

The PHQ-9 score (median and IQRs) was 7.0 (4.5–11.0) in MRKH patient group while 6.0 (3.0–9.0) in the age-matched control group, the former being significantly higher than the latter (*P* = 0.015). Altogether, 75.2% (106/141) of patient group and 61.2% (109/178) of control group suffered from depressive symptoms (PHQ-9 score ≥ 5). Among the patient group, 24.8% (35/141) had no symptoms, 41.1% (58/141) had mild symptoms, 20.6% (29/141) had moderate symptoms, 8.5% (12/141) had moderately severe symptoms and 5.0% (7/141) had severe symptoms. While in the control group, 38.8% (69/178) had no symptoms, 37% (66/178) had mild symptoms, 18.6% (33/178) had moderate symptoms, 3.4% (6/178) had moderately severe symptoms and only 2.2% (4/178) had severe symptoms. Using 10 as a cutoff point, 34.0% (48/141) of patients with MRKH syndrome were allocated to the depressive group (Fig. 1).

According to the last question in the PHQ-9, 11 (7.8%) patients with MRKH syndrome admitted that they had self-injury or suicidal ideas more than half of the time in the past 2 weeks, and 5 (3.5%) patients had these ideas almost every day.

### Depressive scores in different age groups of patients with MRKH syndrome

**Fig. 2:**
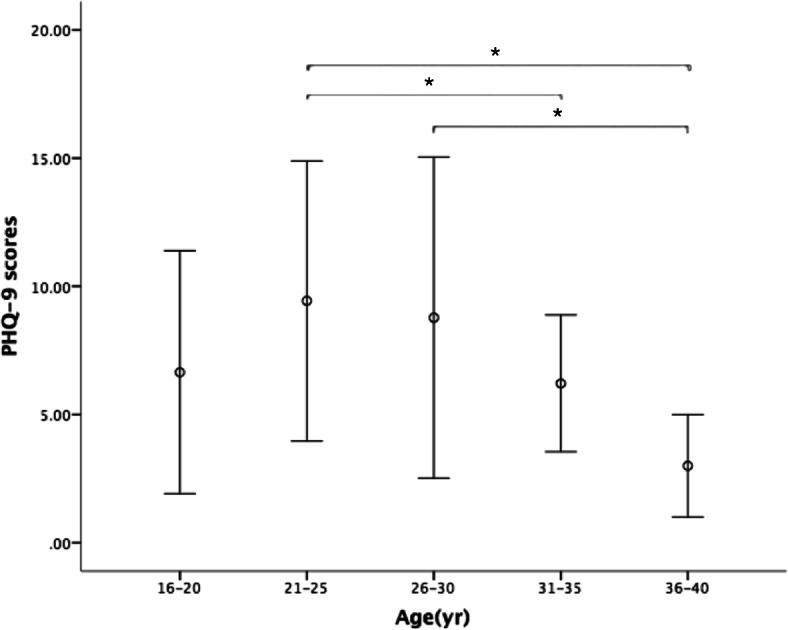
Depressive scores in different age groups. ANOVA methods were used to compare PHQ-9 scores in different age groups. Participants in the 21–25 yr group scored significantly higher than those in the 31–35 yr and 36–40 yr groups, and 26–30 yr participants also scored higher than those in the 36–40 yr group. Results are expressed as mean ± SD. * stands for *P* < 0.05

Based on the age span, the patients were divided into 5 age groups: 16–20 yrs., 21–25 yrs., 26–30 yrs., 31–35 yrs. and 36–40 yrs. The ANOVA methods were applied to compare the depressive scores of different age groups. Patients in the 21–25 yrs. group scored significantly higher than those in the 31–35 yrs. (*P* = 0.037) and 36–40 yrs. groups (*P* = 0.009), and 26–30 yrs. patients also scored higher than those in the 36–40 yrs. group (*P* = 0.016) (Fig. 2).

### Factors associated with depressive symptoms in the patient group

Then, a univariate analysis was conducted to compare the differences between the depressive and non-depressive groups in patients with MRKH syndrome (Table 1). Except for education level, the sociodemographic features and the treatment histories showed no significant difference between the two groups. Compared to the non-depressive group, more participants in the depressive group achieved an education level below secondary school (*P* = 0.043) and believed they did not possess good femininity (*P* < 0.001). However, there was no significant difference between these two groups in the attitudes toward offspring (*P* = 0.620). In addition, the N and P scores in the EPQ-RSC were significantly higher (*P* < 0.001, *P* = 0.016), while the E score was significantly lower (*P* = 0.005) in patients with depressive symptoms, indicating that depressive patients with MRKH syndrome were susceptible to neuroticism, psychoticism and introversion.

In terms of sexual function, 71 patients (treatment vs. non-treatment: 51 vs. 20) had no sexual activity in the past 4 weeks. The number of sexual dysfunction and normal sexual function groups were 28 (treatment vs. non-treatment: 22 vs. 6) and 42 (treatment vs. non-treatment: 35 vs. 7) respectively according to the score of CVFSFI. The χ^2^ test showed that the depressive status was significantly different among the three groups (*P* < 0.001). By pairwise comparison, more patients with sexual dysfunction suffered from depressive symptoms than patients with normal sexual function (Bonferroni-corrected *P* value< 0.001) and patients who had no sexual activity (Bonferroni-corrected *P* value = 0.009).

To further identify the predictors of depressive symptoms, except for the factors with *P* < 0.1 in univariate analysis, we also included the clinically relevant variables (age, years since diagnosis and family support) in the stepwise logistic regression. The results suggested that negative self-evaluation of femininity (OR 3.96, 95% CI 1.37–11.46); neurotic personality traits (OR 1.19 95% CI 1.11–1.28) and sexual dysfunction (compared with those who had no sexual activity, OR 4.81, 95% CI 1.24–18.72) were potential risk factors for depressive symptoms in patients with MRKH syndrome (Table 2).

**Table 2:** Factors associated with depressive symptoms according to stepwise logistic regression analysis

Variables	OR	OR (95% CI)	
		Lower	Upper
Age	0.96	0.83	1.11
Years since diagnosis	0.94	0.80	1.10
Education level	0.81	0.25	2.62
Family relationship	1.43	0.44	4.68
Family support received	0.32	0.06	1.77
Negative self-evaluation of femininity	3.96	1.37	11.46
Female sexual dysfunction (compared with sexually inactive women)	4.81	1.24	18.72
Normal sexual function (compared with sexually inactive women)	0.43	0.12	1.52
Neurotic personality	1.19	1.11	1.28
Introversive personality	1.00	0.96	1.05
Psychotic personality	1.02	0.96	1.08

## Discussion

MRKH syndrome is a congenital disease affecting the normal function of sexual intercourse and breeding, as well as the psychological states of patients. Moreover, because the diagnosis is generally made at puberty, its psychological impact is even greater. To investigate the psychological states of patients with MRKH syndrome, we used the PHQ-9, a brief instrument with satisfactory sensitivity and specificity, to evaluate the depressive symptoms of patients.

Our study reported that the depressive score is significantly higher in women with MRKH syndrome (*P* = 0.015) compared with age-matched healthy controls. However, several studies presented negative results in the screening of depressive symptoms in MRKH patients. An assessment of depression in MRKH patients in the previous studies was reviewed in Table 3 [3, 13–16]. In the limited literatures that evaluated the psychological functioning on MRKH patients, Hospital Anxiety and Depression Scale (HADS) was the most commonly used questionnaire to assess depression symptoms. Only one study by Leithner K et al. using PHQ-9 to screen depression symptoms in ten MRKH patients revealed negative result [15]. One study by Cameron IM et al. to assess the psychometric properties of PHQ-9 and HADS for measuring depression severity revealed that PHQ-9 categorized a higher percentage of patients with moderate/severe depression [17]. Considering the importance of the assessment of depression severity, PHQ-9 was chosen to screen the depression symptoms in our study. This may explain the significantly higher depressive score in patients with MRKH syndrome in our study. Besides, the relatively large sample size, high response rate (over 60%) and age-matched control group in our study might also explain the differences in the screening of depressive symptoms from previous studies. The heavier burden of psychological distress in patients with MRKH syndrome in our study again emphasizes the importance and necessity of screening depressive symptoms in this population.

**Table 3:** Overview of the assessment of depression in MRKH patients

Reference	subjects (response rate; age)	instruments	scores on depression between two groups	strengths	limitations
Heller-Boersma et al., 2009, Psychosomatics [3]	patient group: 66, mean age:27.9 ± 1.0 yrs., response rate: 20% (66/355); control group:31 healthy women, mean age: 27.8 ± 1.5 yrs.	The symptom checklist (SCL-90-R); The Rosenberg Self- Esteem Scale (RSE); The Inventory of Interpersonal Problems (IIP-32)	no significant difference	Well-matched comparison group; Widely used questionnaires;	cross-sectional design; small sample size; selection bias (Response rate: 20%)
				Adds to the limited knowledge of psychological functioning in MRKH patients.	
Claudia Gatti et al., 2010, THE JOURNAL OF UROLOGY [13]	patient group:40, mean age:27.8 ± 1.3 yrs., response rate: 93% (40/43); control group: 30 healthy women, mean age: 27.8 ± 1.4 yrs.	BDI (Beck Depression Index); RSES; FSFI; Cohen Test for Life Management Ability (CTLMA)	no significant difference	Well-matched comparison group; Adds to the limited knowledge	cross-sectional design; small sample size; selection bias
Liao et al., 2011, AJOG [14]	patient group: 56, mean age:21.7 (18–52 yrs), response rate:72% (56/87); control group: standardization population.	SF-12; HADS; FSFI; Multidimensional Sexuality Questionnaire (MSQ)	no significant difference	Widely used questionnaires; Adds to the limited knowledge.	cross-sectional design; normative data are from a general population.
Katharina Leithner et al., 2015, PLOS ONE [15]	patient group:10, mean age:36.7 ± 11.1 yrs., response rate: 59% (10/17); control group: 20 healthy women, mean age:25.5 ± 4.21 yrs.	FSFI; PHQ; Brief Symptom Inventory (BSI); World Health Organization Quality of Life Assessment (WHOQoL-BREF)	no significant difference	Adds to the limited knowledge.	cross-sectional design; small sample size
P.T.M. Weijenborg, et al., 2019, Human Reproduction [16]	patient group: 54, mean age:39.2 ± 13.8 yrs. response rate: 85%(54/63); control group: 79 healthy women, mean age: 36.7 ± 11.1 yrs.	SCL-90; HADS; RSES; FSFI; Female Sexual Distress Scale (FSDS); Female Genital Self-Image Scale (FGSIS); Maudsley Marital Questionnaire (MMQ)	no significant difference	Well-matched comparison group; Widely used and comprehensive questionnaires;	cross-sectional design; selection bias; small sample size;
				Adds to the limited knowledge.	

In this study, the results showed that patients in their 20s suffered more severe depressive symptoms than older groups. Women in their 20s generally start to face sexual experiences, marriage and breeding the next generation. These issues are precisely the problems faced by MRKH patients due to the anomalies caused by this syndrome. Therefore, it is rational that the patients at this stage suffered substantial depression. For individuals at young adult stages, depression leads to interpersonal conflicts, academic underperformance, low self-esteem and even suicide. Thus, more attention and concern should be given to MRKH patients in young adulthood.

Doubts about their roles as females were prevalent in these patients. These women experienced a loss of sexual and social roles following diagnosis. Appearing normal from the outside, some patients struggled to interpret themselves in a stable way, alternating between female-not female polarities [18]. Our study noted that the negative self-evaluation of femininity was a potential risk factor for depressive symptoms. Similarly, Heller-Boersma et al. presented a psychological model that could support our findings, suggesting that the core issue in MRKH was a threat to a patient’s sense of themselves as fully functioning women [19]. Attempting to reduce the sense of threat to femininity, a range of maladaptive cognitive and behavioral strategies emerged.

We also found that neurotic personality might contribute to depressive symptoms in patients with MRKH syndrome. Personality traits are fundamental for the perception of life events. Neurotic people are more vulnerable to negative emotions under psychological distress. As mentioned above, a diagnosis of MRKH syndrome leads to enormous emotional burden. Hence, neurotic patients may develop negative emotions more easily than patients with higher emotional stability. Moreover, Bargiel has proven that MRKH patients score higher in neuroticism compared to healthy women [2], indicating a stronger tendency for MRKH patients to develop negative psychological symptoms, which also reminds us of the importance of screening the psychological states of these patients.

A systematic review by Atlantis E and Sullivan T revealed that sexual dysfunction shares a well-established bidirectional relationship with depression. Sexual dysfunction increases the risk of depression by 130–200%, while depression is associated with a 50–70% increased risk of sexual dysfunction [20]. This is consistent with our finding that patients with sexual dysfunction were more vulnerable to depressive symptoms.

Interestingly, we found that treatment was not a potential risk or protective factor of depressive symptoms in patients with MRKH syndrome. This finding implies that the negative psychological impact on women is long lasting, although treatment may allow patients to have neovagina and successful sexual intercourse. This idea is supported by several previous studies. Ismail Pratt et al. indicated that the depressive symptoms were not altered significantly in MRKH patients after nonsurgical treatment [21]. Djordjevic et al. drew similar conclusions from patients undergoing vaginoplasty [22]. However, group cognitive-behavioral treatment intervention significantly improved psychological outcomes in MRKH [19]. In addition to treatments to normalize the anatomy of the vagina, psychotherapy should be considered as an independent treatment for these patients. This perception was also suggested by the latest guidelines of MRKH syndrome. The American College of Obstetricians and Gynecologists recommended that all patients with Mullerian agenesis should be offered psychological counseling and encouraged to connect with peer support groups in addition to intervention to address the functional effects of genital anomalies [23].

Another interesting observation was that seven non-treated patients had normal sexual function in the study. One reason might be that they were already sexually active and had obtained a vagina of certain length by intercourse at the time of our study. Two previous studies have already reported a successful approach to achieve normal vaginal depth by sexual intercourse in MRKH patients [24, 25]. Another reason might be that except for vaginal intercourse, anal (or oral) sexual activity or non-penetrative sex also played important parts in these patients’ sexual life. An investigation of the overall sexual function in a cohort of North American women shows that in addition to vaginal intercourse (62%), external stimulation from the partner (48%) or themselves (37%) were also important trigger of orgasm [26]. These may explain that despite of their shortened vagina, these seven non-treated patients could also have normal sexual function.

To our knowledge, this study included the largest sample size to screen for depressive symptoms in patients with MRKH syndrome. Moreover, given the confidential feature of this topic, we used the online and anonymous survey to achieve a satisfactory response rate. Though rare, hundreds of thousands of women might suffer from MRKH syndrome. However, the psychological evaluation and intervention towards these patients are quite limited. The study results add new data to the very limited knowledge about psychosexual functioning of women with MRKH syndrome and are of importance for more adequate counseling and treatment of these women.

However, our study had several limitations. First of all, due to the characteristics of the cross-sectional study, we could not determine the causality between depressive symptoms and their associated factors. Moreover, self-rating questionnaires were used in our study, without diagnostic function. In addition, due to the characteristics of anonymous study, we could only analyze the information offered in the questionnaire. The clinical information of respondents during long-term follow-up wasn’t included in the study. We recommend further prospective studies with large sample sizes and diagnostic evaluation to identify the risk factors for depressive symptoms in MRKH syndrome.

## Conclusions

Three-quarters of MRKH patients show depressive symptoms, and one-third of these individuals are at moderate to severe level. Negative self-evaluation of femininity, neurotic personality and female sexual dysfunction were associated with depressive symptoms. These findings underscore the importance of evaluating the psychological states of MRKH patients. To relieve patients from severe psychological impact, psychotherapy should be treated as an independent treatment for these patients.

## Data Availability

The datasets used and analyzed during the current study are available from the corresponding author on reasonable request.

## References

[1] Herlin M, Bjørn A-MB, Rasmussen M, Trolle B, Petersen MB (2016). Prevalence and patient characteristics of Mayer–Rokitansky–Küster–Hauser syndrome: a nationwide registry-based study. Hum Reprod.

[2] Bargiel-Matusiewicz K, Kroemeke A (2015). Personality traits and coping styles in women with Mayer-Rokitansky-Küster-Hauser syndrome. Arch Med Sci.

[3] Heller-Boersma JG, Schmidt UH, Keith ED (2009). Psychological distress in women with Uterovaginal agenesis (Mayer-Rokitansky-Küster-Hauser syndrome, MRKH). Psychosomatics..

[4] Laggari V, Diareme S, Christogiorgos S, Deligeoroglou E, Christopoulos P, Tsiantis J (2009). Anxiety and depression in adolescents with polycystic ovary syndrome and Mayer-Rokitansky-Küster-Hauser syndrome. J Psychosom Obstet Gynecol.

[5] Wagner A, Brucker SY, Ueding E, Gröber-Grätz D, Simoes E, Rall K (2016). Treatment management during the adolescent transition period of girls and young women with Mayer-Rokitansky-Küster-Hauser syndrome (MRKHS): a systematic literature review. Orphanet J Rare Dis.

[6] Ernst ME, Sandberg DE, Keegan C, Quint EH, Lossie AC, Yashar BM (2016). The lived experience of MRKH: sharing health information with peers. J Pediatr Adolesc Gynecol.

[7] Wang W, Bian Q, Zhao Y, Li X, Wang W, Du J (2014). Reliability and validity of the Chinese version of the patient health questionnaire (PHQ-9) in the general population. Gen Hosp Psychiatry.

[8] Kroenke K, Spitzer RL, Williams JBW (2001). The PHQ-9: validity of a brief depression severity measure. J Gen Intern Med.

[9] Manea L, Gilbody S, McMillan D (2015). A diagnostic meta-analysis of the patient health Questionnaire-9 (PHQ-9) algorithm scoring method as a screen for depression. Gen Hosp Psychiatry.

[10] Qian M, Wu G, Zhu R, Zhang S (2000). A revised version of Eysenck personality questionnaire short form scale Chinese version (EPQ-RSC). Aust J Psychol.

[11] Rosen C, Brown J, Heiman S, Leib R (2000). The female sexual function index (FSFI): a multidimensional self-report instrument for the assessment of female sexual function. J Sex Marital Ther.

[12] Ma J, Pan L, Lei Y, Zhang A, Kan Y (2014). Prevalence of female sexual dysfunction in urban chinese women based on cutoff scores of the Chinese version of the female sexual function index: a preliminary study. J Sex Med.

[13] Gatti C, Del Rossi C, Lombardi L, Caravaggi F, Casolari E, Casadio G (2010). Sexuality and psychosocial functioning in young women after Colovaginoplasty. J Urol.

[14] Liao L-M, Conway GS, Ismail-Pratt I, Bikoo M, Creighton SM (2011). Emotional and sexual wellness and quality of life in women with Rokitansky syndrome. Am J Obstet Gynecol.

[15] Leithner K, Naderer A, Hartung D, Abrahamowicz C, Alexopoulos J, Walch K (2015). Sexual and psychosocial functioning in women with MRKHS after Neovaginoplasty according to Wharton-Sheares-George: a case control study. Fischer G, editor. PLoS One.

[16] Weijenborg PTM, Kluivers KB, Dessens AB, Kate-Booij MJ, Both S (2019). Sexual functioning, sexual esteem, genital self-image and psychological and relational functioning in women with Mayer-Rokitansky-Küster-Hauser syndrome: a case-control study. Hum Reprod.

[17] Cameron IM, Crawford JR, Lawton K, Reid IC (2008). Psychometric comparison of PHQ-9 and HADS for measuring depression severity in primary care. Br J Gen Pract.

[18] Holt R, Slade P (2003). Living with an incomplete vagina and womb: an interpretative phenomenological analysis of the experience of vaginal agenesis. Psychol Health Med.

[19] Heller-Boersma JG, Schmidt UH, Edmonds DK (2007). A randomized controlled trial of a cognitive-behavioural group intervention versus waiting-list control for women with uterovaginal agenesis (Mayer–Rokitansky–Küster–Hauser syndrome: MRKH). Hum Reprod.

[20] Atlantis E, Sullivan T (2012). Bidirectional association between depression and sexual dysfunction: a systematic review and meta-analysis. J Sex Med.

[21] Ismail-Pratt IS, Bikoo M, Liao L-M, Conway GS, Creighton SM (2007). Normalization of the vagina by dilator treatment alone in complete androgen insensitivity syndrome and Mayer-Rokitansky-Kuster-Hauser syndrome. Hum Reprod.

[22] Djordjevic ML, Stanojevic DS, Bizic MR (2011). Rectosigmoid Vaginoplasty: clinical experience and outcomes in 86 cases. J Sex Med.

[23] Committee Opinion No ACOG (2018). 728: Müllerian agenesis. Obstet Gynecol.

[24] Moen MH (2014). Vaginal agenesis treated by coital dilation in 20 patients. Int J Gynaecol Obstet.

[25] Herlin M, Bay Bjørn A-M, Jørgensen LK, Trolle B, Petersen MB (2018). Treatment of vaginal agenesis in Mayer-Rokitansky-Küster-Hauser syndrome in Denmark: a nationwide comparative study of anatomical outcome and complications. Fertil Steril.

[26] Shaeer O, Skakke D, Giraldi A, Shaeer E, Shaeer K (2020). Female orgasm and overall sexual function and habits: a descriptive study of a cohort of U.S. women. J Sex Med.

